# Preclinical assessment of rodent jumping power with a novel electrical stimulation-assisted device

**DOI:** 10.1038/s41598-023-44748-6

**Published:** 2023-10-13

**Authors:** Seung-Rok Kim, Ey-In Lee, Yongnyun Kim, Sang Wouk Cho, Namki Hong, Yumie Rhee, Jin-Woo Park

**Affiliations:** 1 Department of Materials Science and Engineering, Yonsei University, 50 Yonsei-ro, Seodaemun-gu, Seoul, 03722 Korea; 2 Yonsei University Health System, Seoul, 03722 Korea; 3 Department of Integrative Medicine, Yonsei University College of Medicine, Seoul, Korea; 4 Institue for Innovation in Digital Healthcare (IIDH), Severance Hospital, Seoul, Korea; 5 Department of Internal Medicine, Severance Hospital, Endocrine Research Institute, Yonsei University College of Medicine, Seoul, 03722 Korea

**Keywords:** Animal biotechnology, Engineering

## Abstract

Sarcopenia is a progressive loss of muscle mass and strength that is associated with increasing the risk of falls, musculoskeletal diseases, and chronic metabolic diseases. However, the animal models adopted to study sarcopenia face limitations since the functional tests conducted on human cannot be directly adapted to animals because the animals do not follow instructions. Moreover, current preclinical research tools for muscle function assessment, such as the rotarod, grip strength, and treadmill, have limitations, including low-intensity simple movements, evaluator subjectivity, and limited power indicators. Hence, in this study, we present a new jumping-power assessment tool in a preclinical rodent model to demonstrate muscle functions. To overcome the light weight and command issues in the rodent model, we developed an electrical stimulation-assisted jump power assessment device. Precisely, the device utilizes a load cell with a 0.1 g resolution and a 50 points/s data acquisition rate to capture the short period of the mouse jump. Additionally, interdigitated electrodes are used to electrically stimulate the mice and make them jump. While our primary focus in this article is the validation of the newly developed jump power assessment device, it is worth noting that this tool has several potential utilities. These include the phenotypic comparison of sarcopenia models, the exploration of muscle function reduction mechanisms, muscle function-related blood biomarkers, and the evaluation of drug intervention effects.

## Introduction

Sarcopenia, a progressive loss of muscle mass and strength typically associated with aging, is a significant health concern in aging societies worldwide^1–3^, increasing the risk of falls, musculoskeletal diseases, chronic metabolic diseases, and mortality rates^4^. In addition, several factors, such as hormonal changes^5^, a sedentary lifestyle^6^, inadequate protein intake^7^, and chronic illnesses^8^, can cause sarcopenia. More particularly, sarcopenia is a significant health concern that is anticipated to become more prevalent as the population ages^3^. Nevertheless, the early identification and adequate treatment of sarcopenia are vital to prevent further muscle loss. Generally, several methods may be used to diagnose sarcopenia in humans, including physical examination, functional assessments, and imaging techniques^9^. More specifically, the combination of these different methods can comprehensively evaluate muscle health and help diagnose sarcopenia. Furthermore, there is a need for effective interventions and biomarkers for sarcopenia, as well as more refined and validated preclinical tools to evaluate muscle function and pathology^1^.

Among the available diagnosis schemes, there is a growing interest in the adoption of functional tests^10^, such as the timed-up-and-go test, the 6-min walk test, and the stair climb power test, to evaluate mobility and physical functions. This is because they provide a more holistic assessment of muscle function than the traditional measures of muscle mass and strength. Specifically, functional tests can evaluate how well an individual can perform daily activities, which is essential to maintaining independence and a quality of life. In addition, the countermovement jump is another functional assessment tool that can diagnose sarcopenia in human patients^11,12^. Specifically, by measuring the force and power generated during a jump, information regarding the muscle function and power can be obtained, which is a critical component of diagnosing sarcopenia.

To prevent or treat sarcopenia, a resistance training program and adequate protein intake are essential^13^. However, many older adults may struggle to participate in exercise programs due to physical limitations, comorbidities, or the lack of access to appropriate facilities or trained professionals. Therefore, there is a need for effective pharmacological interventions for sarcopenia, but to date, no drugs have been approved specifically for this indication. Moreover, there is a need for reliable biomarkers for sarcopenia since the current diagnostic criteria are based primarily on the presence of low muscle mass and function, which may not accurately reflect the underlying pathophysiology of the condition. In particular, biomarkers that can detect early changes in the muscle mass, strength, or metabolism may help identify individuals at risk of sarcopenia and monitor the effectiveness of medical interventions^14^. At present, research concerning the mechanisms of sarcopenia and the development of effective interventions and biomarkers is an active area of investigation.

Generally, animal models, particularly rodents, are commonly used to study the pathophysiology of sarcopenia and consequent potential test interventions^15^. Specifically, biological muscle function assessment is a critical outcome variable in preclinical research to validate sarcopenia treatments and biomarkers. However, the existing preclinical research tools for muscle function assessment, such as the rotarod, grip strength, hanging wire, and treadmill, have limitations involving measuring low-intensity simple movements, learning effects, evaluator subjectivity, and limited power indicators^16^. Additionally, while the measurement of force and torque output through direct electrical stimulation of muscles can exhibit the muscle quality^17–19^, they are invasive methods which can pose constraints on the extent of further research. Therefore, it is necessary to develop a new muscle functional assessment tool that can overcome these limitations and accurately evaluate muscle function in preclinical research. However, there is a need for more sophisticated preclinical tools to evaluate muscle function and biomarkers in these models.

In this study, we developed a novel electrical stimulation-assisted jump power assessment device in a preclinical rodent model to determine muscle functions. More specifically, countermovement jumping force measurement in rodent models is challenging to obtain because the mice are lightweight with a mass that is typically less than 100 g. As a result, the jumps cannot be recorded as intended. Thus, to overcome this challenge, we adopted a load cell with 0.1 g resolution and a 50 point/s data acquisition rate in the cage to capture the short period of mouse jumping. Additionally, a pair of electrodes with an interdigitated pattern is utilized to electrically stimulate the skin surface of the mice, which can annoy the mice and cause them to jump. Finally, we have provided a calibration process for the error induced by the data fluctuations due to the restless mice. Overall, we expect the potential adoption of the developed preclinical rodent jumping power assessment tool for the phenotypic comparison of sarcopenia models, the exploration of muscle function reduction mechanisms, the exploration of muscle function-related blood biomarkers, and the evaluation of drug intervention effects.

## Results

### Countermovement jump assessment setup to obtain maximum jumping power

**Figure 1:**
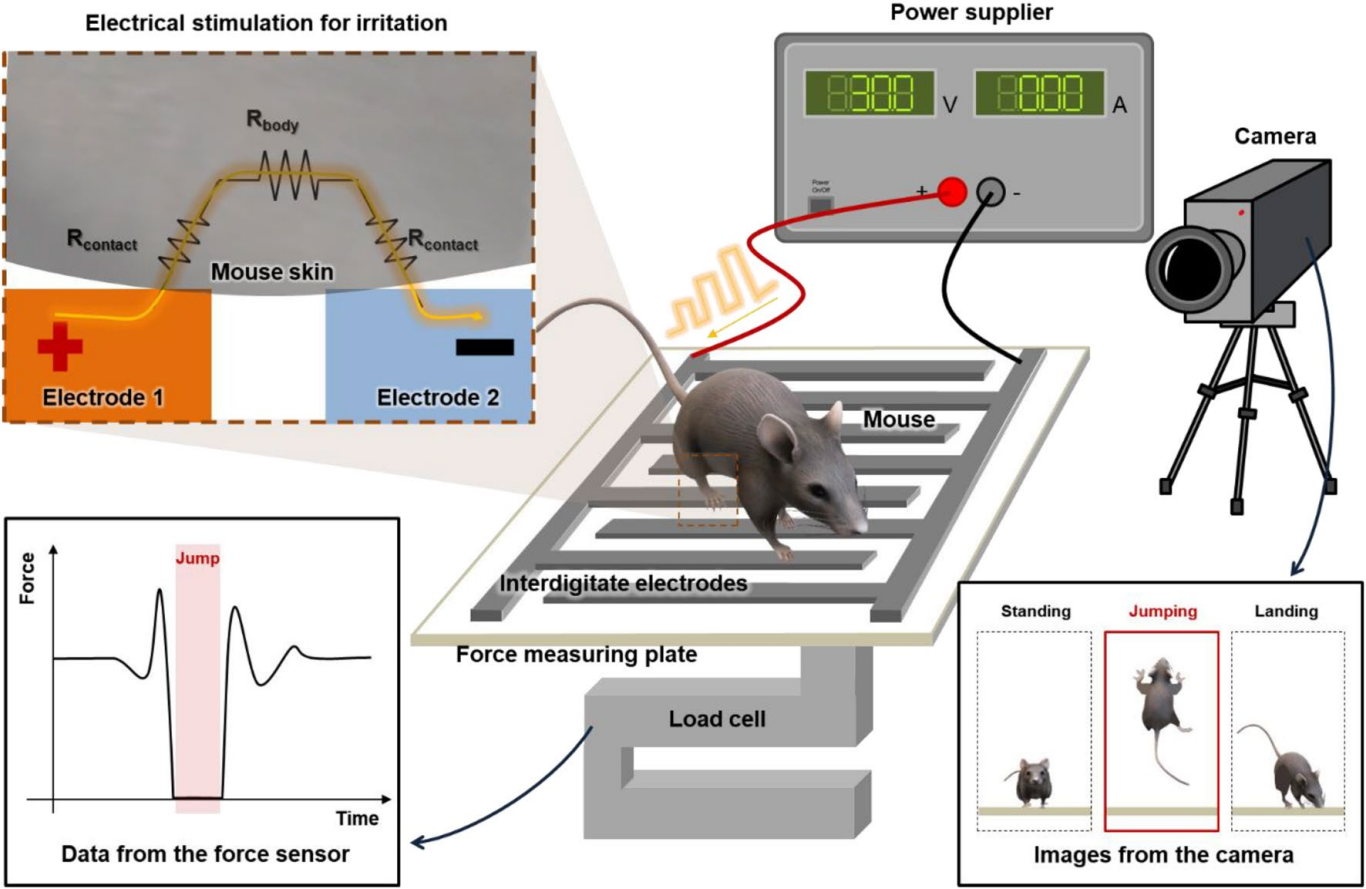
The schematic illustration of the countermovement jump assessment tool for the rodent model consists of a power supply, weight-measuring load cell, and camera. Specifically, the inset image with a dashed box is the mechanism of the electrical stimulation for irritation under the electrical field with the interdigitated electrodes. Additionally, the inset image in the solid box from the load cell is the generic force variation data from the mouse jumping. The other inset image in the solid box from the camera is the visual identification of the jumping mouse.

In a rodent model, we cannot guide a mouse to jump to measure its maximum countermovement jumping power. In addition, not all jumps can be successfully measured. Moreover, their lightweight nature (less than 100 g) makes capturing the force variations for the peak jumping power even more difficult. Therefore, the setup to assess the countermovement jumps of rodent models should include the weight-measuring force sensing part, the power-supplying electrical stimulator part, and the visually recording camera part (Fig. 1).

The crucial point details that the measurement of the peak jump force should be obtained when the mouse jumps in the air, but it is uncommon for mice to exhibit such jumps. However, in the countermovement jump assessment tool, we can irritate the mouse with electrical stimulation and allow them to jump to escape from the electrical stimulation on the electrodes. In particular, the paired electrodes with the interdigitated pattern on the force measuring plate can function as both the anode and cathode, applying the voltage to the superficial surface of the mouse. Specifically, when the mouse contacts the cathodic and anodic electrodes, the whole body can be treated as a total resistor with two contact resistances (R_contact_) and one body resistance (R_body_). Interestingly, existing research shows that dry skin is a dielectric with a high total skin impedance of over 10^6^ Ω at 1 Hz^20^. More precisely, when the dielectric skin is under a direct current (DC) electrical field of less than the dielectric strength of the skin, only the displacement current can store the surface charges until the voltage is supplied^21^. This current can induce irritation. However, electrocution can also occur when the electrical field is sufficiently high to penetrate the skin via overvoltage electrons. Therefore, it is necessary to optimize the voltage output for both irritation and safety.

Furthermore, the load cell under the force measuring plate detects the continuous force variation of the mouse due to movement, such as jumping or leaning on the wall. In response to irritating electrical inputs, a jumping mouse creates a generic force variation profile with a zero-force jump part (Fig. 1). Simultaneously, the mouse relaxing or jumping in the cage can be recorded on the camera. Particularly, from the real-time or captured images, we can determine the success of the jumps. More precisely, the whole body of the mouse, including the tail, should be above the ground. Additionally, the mouse should not collide with any wall or the ceiling of the cage to avoid deteriorating the jumping energy. After selecting the successful jump from the camera, we can calculate the muscle function-related power from the force variation profile.

**Figure 2:**
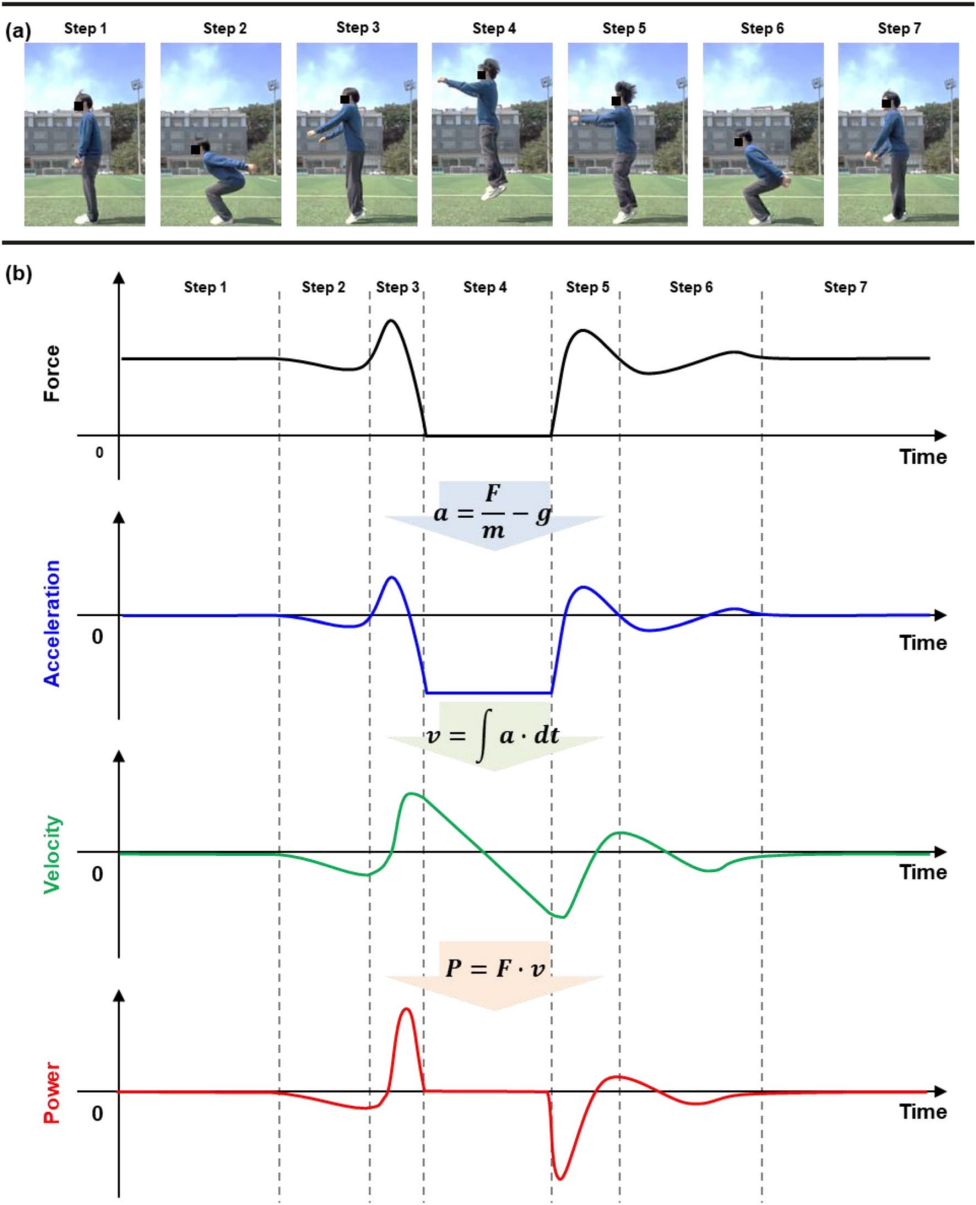
(**a**) The captured sequential seven steps of a human participant starting from a standing position and then vertically jumping, including standing (Step 1), countermovement (Step 2), push-off (Step 3), flight (Step 4), landing (Step 5), recovery (Step 6), and standing (Step 7). (**b**) The generic data calculation process of the ground impacting *P* from the countermovement jump from the *F* profile.

We considered a system in which a human participant starts from a standing position and vertically jumps to calculate the ground-impacting power from the force variation. As presented in Fig. 2a, a human vertical jump occurs in a continuous and sequential seven-step process involving standing, countermovement, push-off, flight, landing, recovery, and standing. In the countermovement (Step 2), a person standing on the ground (Step 1) rapidly flexes the knees and hip, storing potential energy in the muscles of the lower limbs. Particularly, when the person extends their knees and hip to push-off the ground (Step 3), the potential energy is converted into kinetic energy to propel the body upward. During the flight off the ground in Step 4, the person reaches the maximum height from the ground and returns to the ground. In Steps 5 and 6, the person lands on the ground and recovers, absorbing the impact with their legs. In the last step, the person returns to the starting position.

Furthermore, the energy transformations among kinetic energy, potential energy, elastic energy, and chemical-potential energy are used to induce kinematic variations. For example, during countermovement, the muscles are stretched, and thus, potential energy is stored in the elastic elements of the muscle–tendon unit. Consequently, the potential energy is converted into kinetic energy during the push-off, where the stored energy is released to produce force and acceleration. Additionally, the muscle fibers use chemical potential energy in the form of adenosine triphosphate (ATP) to perform work in both steps.

Furthermore, we can interpret this energy exchange rate during the countermovement jump to muscle functioning by measuring the ground-impacting power (*P*). To be specific, we can calculate the power from the gravitational force (*F*) on the ground as in Fig. 2b. The mass of the subject (*m*_0_) is the initial *F* divided by the gravitational constant (*g*). In the system with a constant *m*_0_, the *F* variation from the initial standing state (∆*F*) can induce acceleration (*a*), as shown in Eq. (1):

1 $$a = \Delta F/m_{0} = \, (F{-}m_{0} \cdot g)/m_{0} = F/m_{0} {-}g.$$

By continuously changing *a* during the jump, we can calculate the velocity (*v*) of the subject as time (*t*) advances, as shown in Eq. (2):

2 $$v = \smallint a \cdot {\text{d}}t.$$

Using *F* and *v*, we can evaluate the transferred work in the form of *P*, as shown in the following Eq. (3):

3 $$P = F \cdot v.$$

In the ideal case, there are two *P* peaks during the jump—at the push-off and the landing. Specifically, the two peaks should have opposite signs, because the person can obtain the energy from or provide the energy to the ground during jumping or landing, respectively.

### Countermovement jump assessment tool for rodent models

**Figure 3:**
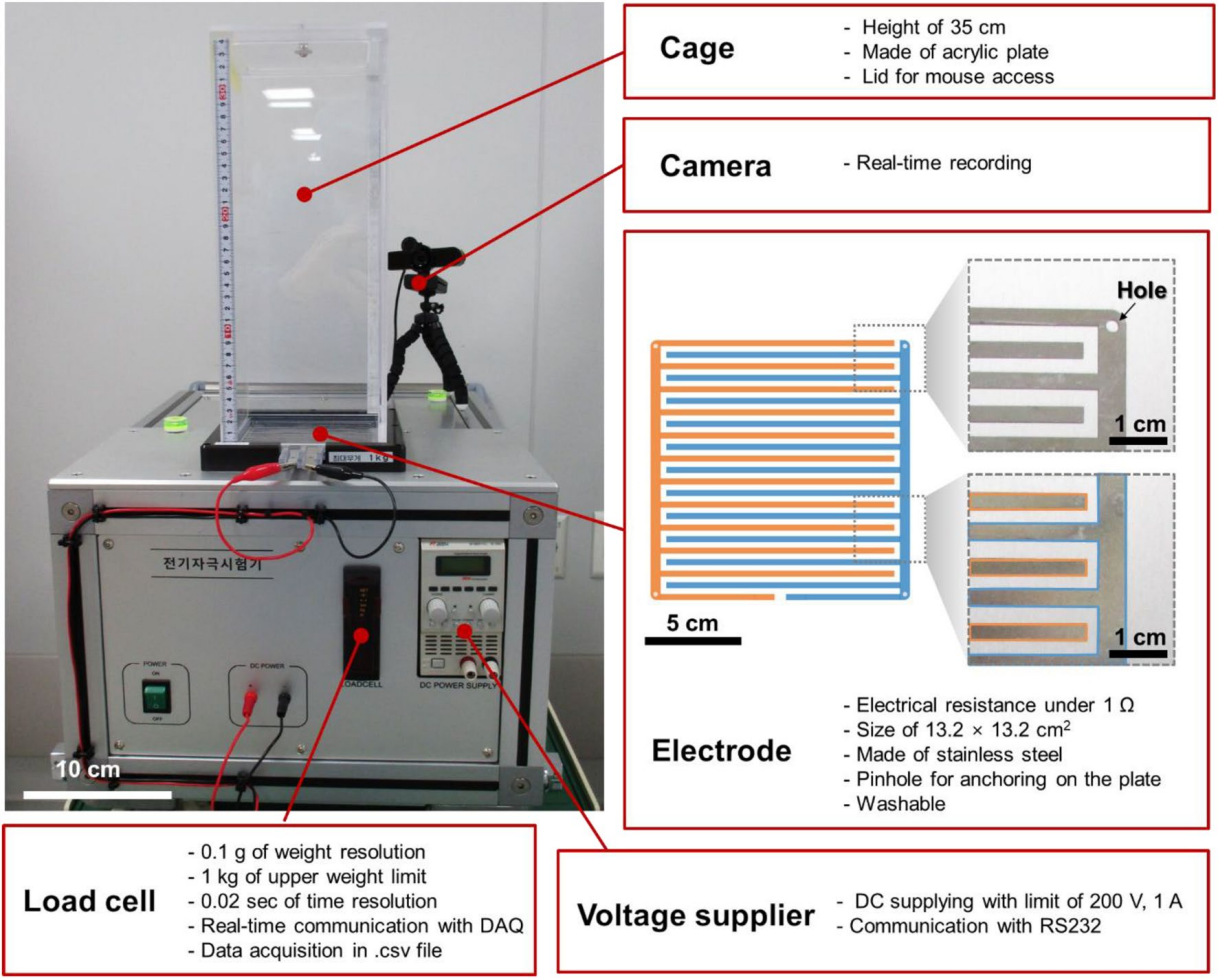
Snapshot image of the countermovement jump assessment tool for the rodent models and the descriptions of the components.

As shown in Fig. 3, the countermovement jump assessment tool for rodent models was fabricated by assembling a weight-measuring load cell and a power supply into a single experimental setup. Additionally, auxiliary interdigitated electrodes, a transparent cage with a lid, and a camera connected to a computer should be mounted on the gadget. Particularly, in this prototype, we exploited the load cell with a 1 kg weight limit and 0.1 g weight resolution. Specifically, the load cell communicates with the data acquisition (DAQ) system in real time to retrieve data points every 0.02 s. More precisely, the recorded data can be saved in an Excel file (.csv) with *a*, *v*, and *P* calculated from *m*. The power supplier can supply direct current (DC) with a limitation of 200 V of voltage and 1 A of current. In addition, the power supply can be controlled by a computer communicating through RS232.

Furthermore, to electrically stimulate superficial surface of the mouse, we opted for the interdigitated pattern as the interface for mouse contact. Specifically, the interdigitated pattern electrodes consist of interlocking fingers with small gaps between the positive and negative electrodes. When a voltage is applied to the electrode pair, the interdigitated pattern creates an electrical field in every small gap along the electrodes. These electrodes exhibited an electrical resistance under 1 Ω. Moreover, since the electrodes were made of stainless steel, they can be reused after washing. Additionally, the patterns were targeted to cover an area of 13.2 cm × 13.2 cm with a gap distance of less than 0.5 cm. In addition, we also made pinholes in the corners of the electrodes to secure the electrodes in the planned position. With the real-time communicating camera, we prepared a transparent, acrylic cage with a height of 35 cm and a lid on the top.

**Figure 4:**
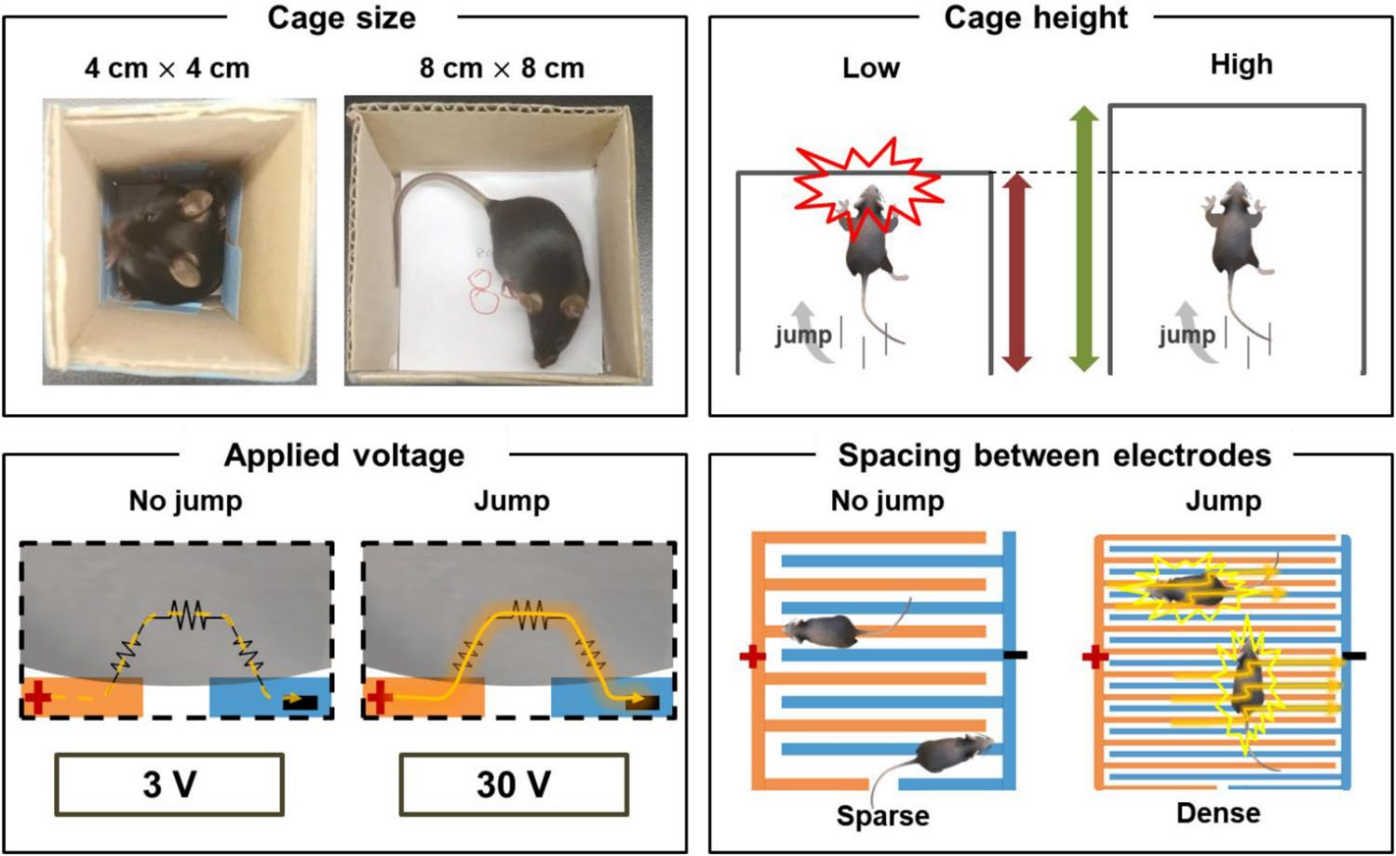
Variables considered for the successful countermovement jump of the rodent models.

Although we successfully fabricated an assessment tool with functions that include electrical stimulation and force measurements, some practical problems occurred when a mouse was placed on the jumping assessment machine. Thus, we will discuss four variables that need to be considered while designing the assessment tool for the successful countermovement jump of the rodent models shown in Fig. 4.

Specifically, when we determine the success of the jumps, *m* should not be affected by the environment, such as the walls or ceiling. Moreover, the cage size should be larger than the size of the mouse to prevent the distribution of its weight along the walls. In addition, the cage height can influence the results when the ceiling restricts the jumping mouse. In addition, the applied voltage should be carefully monitored. As we mentioned in Fig. 1, dry skin is a dielectric and has a high total skin impedance. Hence, there were individually distinctive threshold voltages for the mice. Specifically, in our preliminary test, ten mice jumped on the plate to escape when a voltage of 30 V was applied, whereas a 3 V voltage irritated no mice. Finally, the pattern spacing gap should be considered based on the mouse size. When we made a sparse pattern, the mice learned that standing on a single electrode was safe. Thus, we need to increase the number of finger electrodes to ensure that the mice contact the electrodes.

### Mouse jumping test with the countermovement jump assessment tool

**Figure 5:**
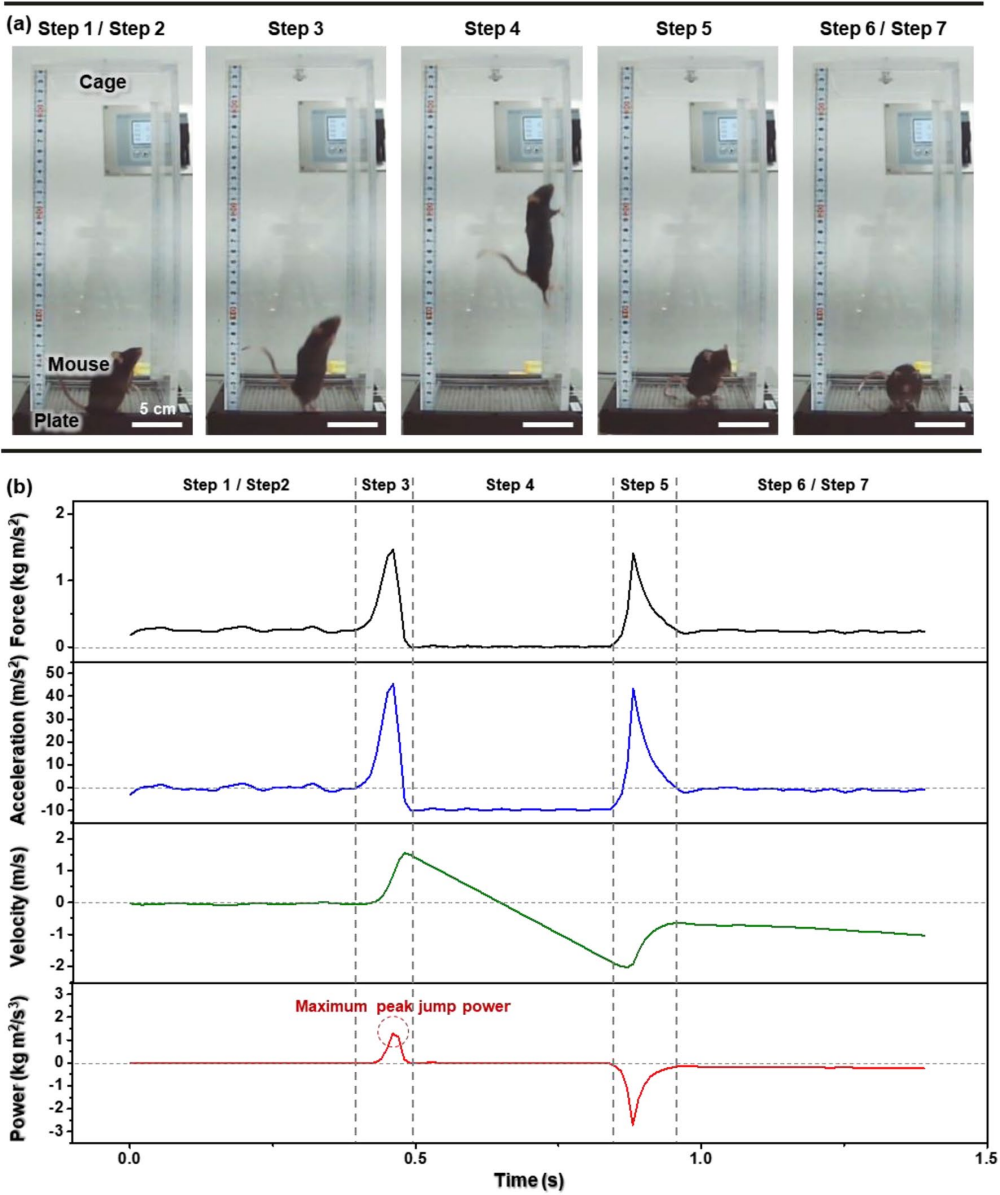
(**a**) Snapshot images of a successful mouse jumping on the force plate under an electrical field. (**b**) The *P* calculation process from the *F* profile during the successful jump.

Adopting the machine designed and fabricated in Figs. 3 and 4, we pre-tested the countermovement jump assessment on the rodent model. Specifically, when we applied 30 V to the interdigitated electrodes, the mouse felt irritated and jumped to escape from the electrical stimulation. As shown in Fig. 5a, we can depict the successful jump from the recorded video with the requirement that the whole-mouse body should be above the ground and match the mouse jumping process to human jumping steps. Compared to the human jumping shown in Fig. 2, we cannot strictly distinguish the mouse's countermovement (Step 2) from the starting position (Step 1). Additionally, the energy-absorbing recovery step (Step 6) was difficult to determine after landing (Step 5). However, the push-off, flight, and landing steps were certainly captured.

Furthermore, we mounted the real-time calculation program based on the kinematic Eqs. (1–3) on the countermovement jump assessment tool. Specifically, continuous but discrete *F* values with a rate of 50 points/s were numerically transformed to *a*, *v*, and *P*. For data processing, it is noteworthy that when *t*_n_, *a*_n_, *v*_n_, and *P*_n_ are n-th values (n is a natural number) of *t*, *a*, *v*, and *P* in the dataset (*v*_1_ and *P*_1_ do not exist), the kinematic Eqs. (1–3) can be changed to the following Eqs. (4–6), respectively:

4 $$a_{{\text{n}}} = F_{{\text{n}}} /m_{0} {-}g.$$

5 $$v_{{\text{n}}} = \sum a_{{{\text{n}} - 1}} \cdot \Delta t = \sum a_{{{\text{n}} - 1}} \times 0.02\quad ({\text{n}} \ge 2)$$

6 $$P_{{\text{n}}} = F_{{\text{n}}} \cdot v_{{\text{n}}} \quad ({\text{n}} \ge 2).$$

As shown in Fig. 5b, the corresponding *P* calculation was conducted with the abovementioned procedure from the successful jump *t* − *F* dataset. In particular, we can quickly determine the maximum peak jump power (red dashed circle) during the push-off.

Based on this preliminary result, we redefined the success of the jump. Ideally, a successful jump should not include several failures, such as leaning on the wall before the start, hitting the wall during flight, or using the tail to apply support on the plate during the jump. Those cases can cause an unexpected calculation error by changing the initial force value or the involved potential energy. However, as shown in the flight step (Step 4 in Fig. 5a), the mouse jumped toward the wall in most cases. Total energy loss by hitting the wall induced a continuous *v* change after landing, even under a constant force. Considering the jump toward the wall as the natural trait of the mouse, we should classify the jump hitting the wall during flight as a success and use only the maximum peak jump power value during the push-off.

### Analytical method for data analysis

**Figure 6:**
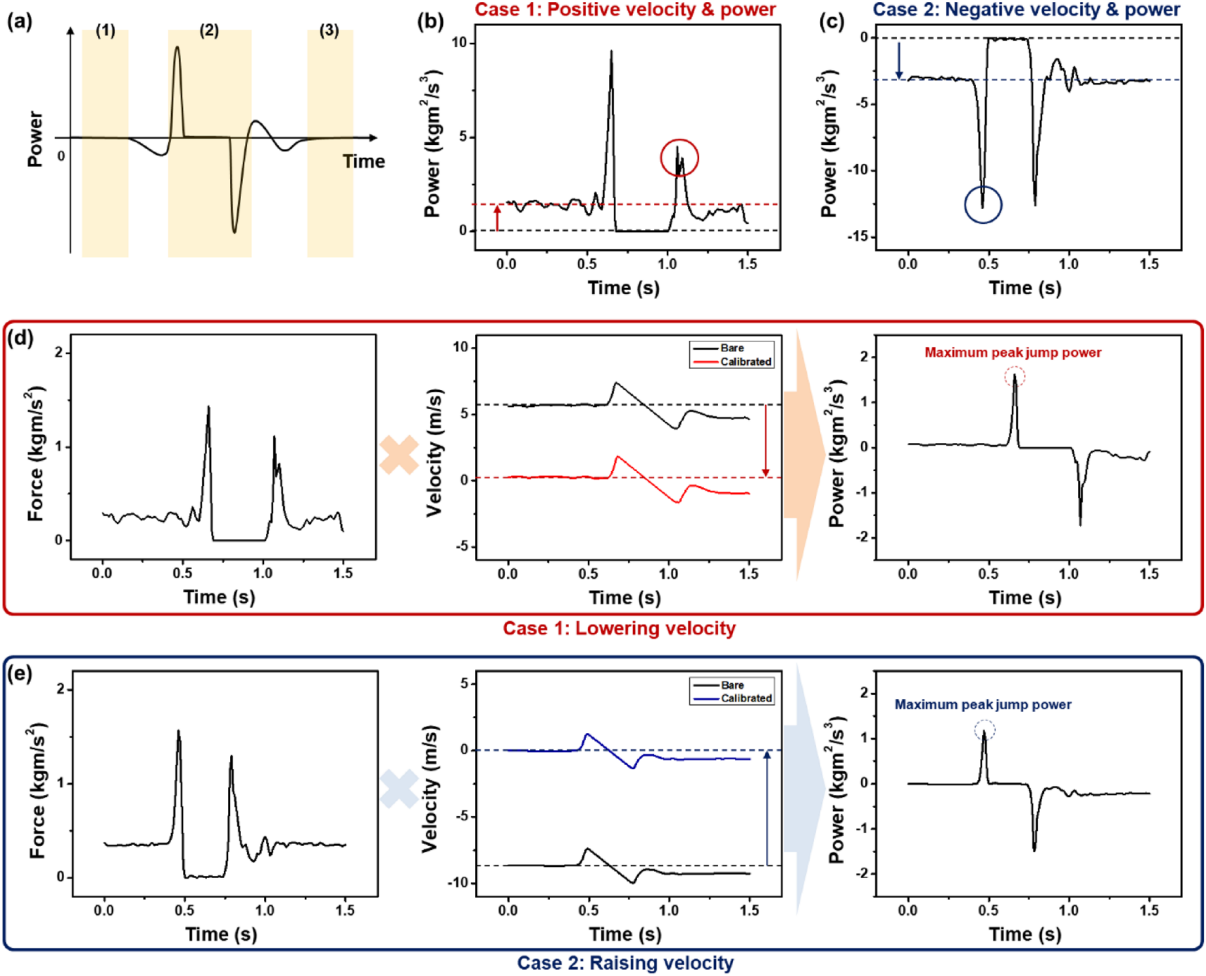
(**a**) Ideal jump *P* profile with variation only in the jumping and landing tasks. Two error cases were identified under the repetitive test: (**b**) Positive *v* & *P* case and (**c**) Negative *v* & *P* case. (**d**,**e**) After calibrating *v* from the shift error, the maximum peak jump *P* can be calculated with the ideal jump *P* profile.

To confirm that the whole-mouse body was above the ground, we exploited real-time camera images to distinguish successful jumps from failed jumps. However, some signals of the successful jumps had slightly different distortions from the ideal successful jumping case. As illustrated in Fig. 6a, the *P* profile in this ideal case can be divided conspicuously into three parts: (1) before jumping, (2) jumping and landing, and (3) after landing. Specifically, in both Part (1) and Part (3), there should be no energy transfer between the jumping object and the ground with no *P* variation. Additionally, in Part (2), there are sharp energy transfers just before jumping and right after landing. Precisely, the peak *P* before jumping is positive, and the other peak that occurs immediately after landing is negative.

However, we found that the non-ideal cases in Fig. 6b,c are caused by the environmental problem before the jump. Specifically, Case 1 shows the baseline positively shifted from zero and two positive-only peaks in the *P* graph during the jumping and landing tasks. Meanwhile, Case 2 shows a negatively shifted baseline and two negative-only peaks in the *P* graph during the jumping and landing tasks. Additionally, because *P* can be calculated from *v* and *F* in Eq. (3) and *F* is the value from the load cell, obtaining *v* may be difficult. As we suspected, the *v* values in Cases 1 and 2 have been shifted from the zero baselines in Fig. 6d,e. When we separate the time domain with independent n-paths, we can express the total *v* in Eq. (2) as the following Eq. (7):

7 $$v_{{{\text{total}}}} = \smallint_{{{\text{path}}\;1}} a_{{{\text{path}}\;1}} \cdot {\text{d}}t_{{{\text{path}}\;1}} + \smallint_{{{\text{path}}\;2}} a_{{{\text{path}}\;2}} \cdot {\text{d}}t_{{{\text{path}}\;2}} + \cdots + \smallint_{{{\text{path}}\;{\text{n}}}} a_{{{\text{path}}\;{\text{n}}}} \cdot {\text{d}}t_{{{\text{path}}\;{\text{n}}}} .$$

In addition, we can re-express the total *v* with the ideal path and error-occurring path as the following Eq. (8):

8 $$v_{{{\text{total}}}} = \smallint_{{{\text{ideal}}}} a_{{{\text{ideal}}}} \cdot {\text{d}}t_{{{\text{ideal}}}} + \smallint_{{{\text{error}}}} a_{{{\text{error}}}} \cdot {\text{d}}t_{{{\text{error}}}} .$$

The linear relationship is the reason why we can calibrate the error by extracting the shifted error values from *v*_total_. As shown in Fig. 6d,e, we calibrated *P* by lowering and raising the baseline of *v* and successfully obtained an ideal jump *P* profile with the maximum peak jump *P* (the red/blue dashed circles in Cases 1 and 2, respectively).

## Discussion

In this work, we successfully demonstrated a novel jumping-power assessment tool in a preclinical rodent model. Specifically, we developed an electrical stimulation-assisted jump power assessment device to overcome the light weight and command issues associated with rodent models. After determining the success of the jump from the camera image, we obtained the ideal maximum peak jump power. Additionally, we proposed a calibration method for the shifted velocity case and proved its accuracy. Particularly, this new tool has various potential applications, such as the phenotypic comparison of sarcopenia models, the investigation of mechanisms related to muscle function reduction, the exploration of muscle function-related blood biomarkers, and the evaluation of the effects of drug interventions.

In the studies of older adults in humans, impairment of countermovement jump power was observed at earlier time points compared to other measures of physical performance such as timed get-up-and-go test, chair rise test, or muscle force measures such as handgrip strength^12,22,23^. Jump power assessment improved fracture risk prediction when added to conventional measures of physical performance and muscle strength by detecting individuals who have impaired physical performance at an earlier stage^24^. These findings, which indicate the value of jump power measurement as a sensitive biomarker of age-related changes in physical performance, can be translated as the potential advantage of countermovement jump power assessment in rodent models of aging and sarcopenia. Natural aging mouse models, at least 18- to 24-month-old mice equivalent to 56- and 69-year-old humans, are the most used models to study sarcopenia^16,25^. However, this model requires a lot of time and resources to build because the impairment of physical performance measurements became evident at least after 18-month-old mice. If the decline of jump power in mice can be effectively detected at an earlier time point, such as between 14 and 18 months, it can be a valuable preclinical model to study the biology of early-stage progression of sarcopenia. Longitudinal, repetitive in-vivo jump power testing is feasible, which can be another strength of this tool to study the effect of exercise/training or pharmacologic intervention as a preclinical model within a short time, especially in the early stage of sarcopenia. A computerized system to calculate the jump power may guarantee more accurate and reproducible results, a major current unmet need in preclinical models for physical performance. Measurement of jump power relative to body weight can be beneficial to detect the efficiency of force generation for a given time independent of muscle mass and body weight, particularly in the high-fat accelerated aging model or sarcopenic obesity model.

However, there are still challenges to be addressed regarding this assessment tool. Specifically, the system currently requires manual operation to control the voltage, lacks a standard threshold for determining the success of a jump, and relies on arbitrary judgment based on camera images to identify the jump in the continuous data flow. As a result, we are currently developing an automated countermovement jump assessment tool to alleviate these issues. More precisely, our ongoing work will involve training the system on voltage inputs to establish a threshold and implementing jump identification and success determination based on quality check logic. Once these challenges are resolved, we will be able to apply this machine to assess the jumping power in several rodent models. This study is reported in accordance with ARRIVE guidelines.

## Methods

### Device specification

In the countermovement jump assessment tool for rodent models, we utilized a commercially available web camera, load cell, and programmable power supply. Plastic cages, stainless steel electrodes, and machine cases were made in-house. The load cell has a 1 kg weight limit and 0.1 g weight resolution. The power supply is a PT200-1 (ODA Technologies, Korea) with a limitation of 200 V of voltage and 1 A of current (200 W).

### Animals

Nine-week-old male C57BL/6 mice were purchased from Joong-ang Experimental Animal Co., Seoul, Korea, and acclimatized in a pathogen-free animal facility for 2 weeks. The mice were kept on a 12-h light–dark cycle at a room temperature of 23 °C ± 2 °C and 55% ± 5% humidity. The mice received standard diets, and water was freely available. Ten mice were participated in the study. All experiments were approved by the Institutional Animal Care and Use Committee of Yonsei University Health System. We confirm that all methods were performed in accordance with the relevant guidelines and regulations of Institutional Animal Care and Use Committee of Yonsei University Health System.

### Mouse jumping test

The animals were placed on the ground reaction force plate and acclimatized for 2 min. The animals were administered an electric shock with an intensity of 30 V until the first jump occurred. The electric shock is immediately stopped after the jumping occurred. Trials without a jump within 20 s of the maximum duration of an electric shock were excluded. Each trial was performed 3 times at 2-min intervals to obtain the best performance.

## Data Availability

The data that support the findings of this study are available from the corresponding author upon reasonable request.
